# Sarcopenia and sarcopenic obesity: do they predict inferior oncologic outcomes after gastrointestinal cancer surgery?

**DOI:** 10.1186/s13741-016-0052-1

**Published:** 2016-10-26

**Authors:** Kimberly L. Mei, John A. Batsis, Jeannine B. Mills, Stefan D. Holubar

**Affiliations:** 1 Dartmouth College, Hanover, NH USA; 2 Dartmouth-Hitchcock Medical Center, Lebanon, NH USA; 3 Geisel School of Medicine at Dartmouth, Hanover, NH USA; 4 The Dartmouth Institute for Health Policy & Clinical Practice, Lebanon, NH USA

**Keywords:** Body composition, Obesity, Oncologic, Sarcopenia, Surgery, Surgical oncology, Colorectal cancer, Pancreatic cancer, Hepatectomy, Malnutrition

## Abstract

Sarcopenia, or loss of skeletal muscle mass and quality, has been studied as part of aging and adverse health outcomes in elderly patients but has only recently been evaluated as a separate condition in cancer patients and important indicator of adverse outcomes. Currently, its definition and method of assessment are still being debated. Sarcopenia within an increasingly obese population has led to a subgroup with sarcopenic obesity, at even higher risk of adverse outcomes. Yet, sarcopenia often goes undiagnosed in these patients, hidden beneath higher body mass index. Identifying sarcopenic and sarcopenic obese subpopulations would allow for more effective treatment plans and potential avoidance of suboptimal outcomes, as well as the chance to intervene and combat these modifiable risk factors. This review will examine available literature on the definition and methods of evaluating sarcopenia and sarcopenic obesity, summarize the effectiveness of sarcopenia and sarcopenic obesity as predictors of outcomes after gastrointestinal cancer surgery, including colorectal cancer resection, liver resection, and pancreatic resection, and outline strategies to minimize the impact of sarcopenia. It is clear that untreated sarcopenia and sarcopenic obesity can be associated with suboptimal post-operative outcomes, especially infections and disease-free or overall survival.

## Background

Sarcopenia, the loss of skeletal muscle mass and quality that occurs as part of natural aging, can be exacerbated by systemic illnesses. Within the last 10 years, this geriatric syndrome has received increasing attention as a possible predictor of adverse outcomes after surgery (Lentine et al. 2011; Mueller et al. 2015). Traditional oncological therapies and perioperative nutritional assessments that focus on weight loss and markers such as serum albumin have overlooked sarcopenia as a determinant of prognosis after oncological surgery. Presently, there is no practical, objective, or easily accessible measure of individual frailty, a general concept that encompasses age-related decline across physiologic systems as well as in psychological and social well-being, resulting in impaired homeostatic reserve (Clegg et al. 2013; Bauer and Sieber 2008; Singh et al. 2014). Sarcopenia, considered a more reproducible signal of frailty, often precedes its development and could more effectively identify patients at higher risk of suboptimal benefit from systemic chemotherapy and surgery (Peng et al. 2011; van Vledder et al. 2012; Buettner et al. 2016).

With the increased prevalence of obesity, those with both sarcopenia and obesity (sarcopenic obesity (SO)) are at higher risk of adverse outcomes, including disability (Baumgartner et al. 2004) and mortality (Batsis et al. 2014a). These associations have not been well investigated in relation to many cancer surgeries. Sarcopenia is often underdiagnosed (Fielding et al. 2011; Prado et al. 2008), as it is not always easily characterized from overall weight loss alone or decreased body mass index (BMI), especially in obese patients (Batsis et al. 2014b). Establishing standard criteria is critical to accurately evaluate and make use of its prognostic abilities.

In this review, we aim to summarize recent literature regarding the impact and effectiveness of sarcopenia and SO as predictors of gastrointestinal (GI) oncologic surgery outcomes, including colorectal cancer resection, liver resection, and pancreatic resection, and promising methods by which these risk factors can be modified.

## Defining sarcopenia, cancer cachexia, and SO

Early definitions of sarcopenia rely upon measures of muscle mass and neglect a functional specification. Evidence suggests the relationship between low muscle mass and adverse outcomes is not linear or direct; rather, they are linked when low muscle mass is associated with muscle weakness (Cruz-Jentoft et al. 2010; Studenski et al. 2014). Recent definitions have evolved to additionally include measures of muscle performance or strength (Table 1).

**Table 1:** Definitions and cutoffs for sarcopenia and sarcopenic obesity assessment

Sarcopenia definition	Study	Functional component of definition	Body composition analysis method	Muscle mass definition	Pros	Cons	Obesity definition	Pros	Cons
ASM index >2 SDs below sex-specific means of Rosetta study reference data (Gallagher et al. 1997)	Baumgartner (Baumgartner et al. 1998)	–	DXA	ASM/m^2^ (Heymsfield et al. 1990)	–	No functional component	% body fat	Measures have been experimentally validated in comparison with BMI (Gallagher et al. 2000) Highly correlated with estimates from DXA (Baumgartner et al. 1998)	–
Low gait speed or low handgrip strength with low muscle mass	Cruz-Jentoft (Cruz-Jentoft et al. 2010)	Gait speed ≤0.8 m/s or >0.8 m/s with handgrip strength below sex-specific cutoffs	–	–	Contains functional component, capturing more of sarcopenia due to poor muscle quality/fat infiltration	–	–	–	–
Low gait speed or inability to rise from chair with low handgrip strength and body mass-adjusted ASM below sex-specific cutoffs	Studenski (Studenski et al. 2014)	Gait speed ≤0.8 m/s or inability to rise from a chair with handgrip strength below sex-specific cutoffs (men <26 kg, women <16 kg)	DXA	ASM/BMI	Contains functional component, capturing more of sarcopenia due to poor muscle quality/fat infiltration Recommendations based on the largest, most diverse samples to have been studied	–	BMI	Most common and widely available measure, easy to evaluate	Inaccurate, fluctuates with changes in both muscle and fat
L3mi below sex-specific cutoffs associated with mortality in cohort obtained through optimum stratification	Prado (Prado et al. 2008), Lieffers (Lieffers et al. 2012)	–	Secondary analysis of CT images (Mitsiopoulos et al. 1998; Shen et al. 2004)	L3mi	–	No functional component	BMI	–	–
	Dello (Dello et al. 2013)								
	Voron (Voron et al. 2015)								
	Levolger (Levolger et al. 2015)								
	van Vledder (van Vledder et al. 2012)						Intra-abdominal fat (Yoshizumi et al. 1999)	Significantly associated with disease-free survival in men undergoing resection of colorectal liver metastases (van Vledder et al. 2012)	–
	Harimoto (Harimoto et al. 2013)						–	–	–
	Lodewick (Lodewick et al. 2015)						% body fat	–	–
L3mi in the lowest sex-specific quartile	Miyamoto (Miyamoto et al. 2015)	–	Secondary analysis of CT images	L3mi	–	No functional component	–	–	–
No specific cutoffs established (lower density reflects more frailty)	Sabel (Sabel et al. 2013)	–	Secondary analysis of CT images	PD	A measure of muscle quality or fat infiltration	No functional component	VF, SFD, TBF, or BMI	VF: risk factor for developing colorectal cancer and significantly associated with increased tumor recurrence in colorectal cancer patients (Moon et al. 2008) SFD: significant predictor of wound infection following colectomy for colon cancer (Sabel et al. 2013) TBF: significant predictor of outcome following colectomy for colon cancer (Sabel et al. 2013)	–
TPA/m^2^ equal to or below cutoff obtained through optimum stratification	Peng (Peng et al. 2011)	–	Secondary analysis of CT images	TPA/m^2^	–	No functional component	BMI	–	–
TPA/m^2^ in the lowest sex-specific quartile	Peng (Peng et al. 2012)	–	Secondary analysis of CT images	TPA/m^2^	–	No functional component	BMI	–	–
	Amini (Amini et al. 2015)								
	Joglekar (Joglekar et al. 2015)								
TPV/m^2^ in the lowest sex-specific quartile	Amini (Amini et al. 2015)	–	Secondary analysis of CT image	TPV/m^2^	Volumetric measure rather than cross-sectional assessment and which may be more accurate at assessing a larger sample of muscle mass	No functional component	BMI	–	–
HUAC in the lowest sex-specific quartile	Joglekar (Joglekar et al. 2015)	–	Secondary analysis of CT image	HUAC	A measure of muscle quality or fat infiltration	No functional component	BMI	–	–

To determine ASM, the sum of lean soft-tissue masses for the arms and legs is computed from CT scans and adjusted by height. To determine L3mi, two consecutive CT images are taken from the L3 to the iliac crest, and cross-sectional areas of the sum of all the muscles in these regions are computed and adjusted by body surface area. To determine PD, CT scans of the left and right psoas muscles at the level of the fourth lumbar vertebrae are used. To determine TPA, measure the cross-sectional area of the right and left psoas muscles from CT images at the level of L3 where both vertebral spinae are clearly visible. To determine TPV, take three manual measurements at the level of L3 on the first slice where both iliac crests were visible to assess a total of 55 cm total psoas length and normalize for height. To determine HUAC, compute (right Hounsfield unit calculation + left Hounsfield unit calculation)/2, where the right Hounsfield unit calculation = (right Hounsfield unit*right psoas area)/(total psoas area) and left Hounsfield unit calculation = (left Hounsfield unit*left psoas area)/(total psoas area) from evaluation of both the right and left psoas at the L3 level

*ASM* appendicular skeletal muscle, *SD* standard deviation, *L3mi* L3 skeletal muscle index, or total skeletal muscle cross-sectional area at the level of the third lumbar vertebrae normalized for stature, *Intra-abdominal fat* total cross-sectional area of visceral adipose tissue, *TPA* total psoas muscle area, measured at the level of the L3, *TPV* total psoas volume, measured at the level of the L3, *HUAC* Hounsfield unit average calculation, measure of radiation attenuation or muscle density and fatty infiltration, measured at the level of the L3, *PD* psoas density or muscle attenuation (average radiodensity), measured at the cross-sectional areas of the left and right psoas muscles at the level of the L4, *VF* visceral fat, visceral anterior-to-posterior distance, or the average distance between the anterior aspect of the vertebra and the linea alba, *SFD* subcutaneous fat distance, or the average distance between the linea alba and the anterior skin along T-12 to L4, *TBF* total body fat or total AP distance, the sum of the SFD and VF

In 2010, the European Working Group on Sarcopenia in Older People established a working clinical definition of sarcopenia as the presence of *both abnormally low muscle mass and low muscle function*, either physical performance or strength, in the form of low gait speed or low handgrip strength (Cruz-Jentoft et al. 2010).

In 2014, the Foundation for the National Institutes of Health alternatively defined sarcopenia as a “differential diagnosis” reached through sequential screening for poor physical function, muscle weakness, and low muscle mass. Low gait speed manifested poor physical performance, and inability to rise from a chair (without using arms) or time required to complete five chair stands were also useful standards. Muscle strength was assessed through unadjusted grip strength and muscle mass via body mass-adjusted appendicular lean mass (Studenski et al. 2014).

Sarcopenia is further distinguished from general cancer-related cachexia, “a multifactorial syndrome characterized by an ongoing loss of skeletal muscle mass (with or without loss of fat mass) that cannot be fully reversed by conventional nutritional support and leads to progressive functional impairment,” which encompasses varying levels of body fat and muscle loss (Dodson et al. 2011; Fearon et al. 2006; Fearon et al. 2011; Fouladiun et al. 2005). Sarcopenia specifically denotes muscle loss (Peng et al. 2012). While cancer cachexic patients experience weight loss and diminished BMI, sarcopenic patients can have normal or increased BMI. As a result, it is still important to screen cancer surgery patients for sarcopenia (Fearon et al. 2011).

During aging and malignancy, lean body mass is lost while fat mass remains constant or increases, possibly leading to SO. SO is defined by subjects who fulfill the combination of criteria for sarcopenia and a given measure of adiposity, such as BMI, waist circumference (WC), computerized tomography (CT)-derived total body fat (TBF), or dual x-ray energy absorptiometry (DXA)/bioelectric impedance analysis (BIA)-derived body fat (Batsis et al. 2013; Lieffers et al. 2012) (Table 1). Metrics of adiposity more precise than BMI, which fluctuates with changes in both muscle and adiposity, may predict cancer outcomes more accurately. Traditional BMI cutoffs have poor diagnostic accuracy when identifying obesity, especially in older populations (Batsis et al. 2016; Romero-Corral et al. 2008).

Previously, decreases in muscle mass were considered largely responsible for muscle weakness and mobility impairment in patients with SO, but current research suggests that deterioration of muscle quality (evidenced by phenomena such as “marbling” or fat infiltration into muscle) is an overlooked contributor (Cruz-Jentoft et al. 2010). Both aging and obesity are associated with decline in muscle quality and higher rates of fat infiltration (Zamboni et al. 2008). Studies find that although obese elderly subjects often acquire a higher absolute muscle mass than non-obese frail and normal counterparts to compensate for body habitus (Visser et al. 2002), they have the poorest muscle quality and strength and, like the non-obese frail, exhibit reduced functional status, aerobic capacity, strength, balance, and gait speed (Villareal et al. 2004). This suggests that sarcopenia and SO may be seriously underdiagnosed, since previous definitions largely ignore muscle quality and the retrospective nature of many existing studies investigating sarcopenia and cancer surgery limits them to criteria relying on muscle mass. Moreover, there remains variability within cutoffs used across studies and uncertainty regarding the applicability of these criteria to all ethnic groups (Studenski et al. 2014).

## Diagnosing sarcopenia and SO

**Fig. 1:**
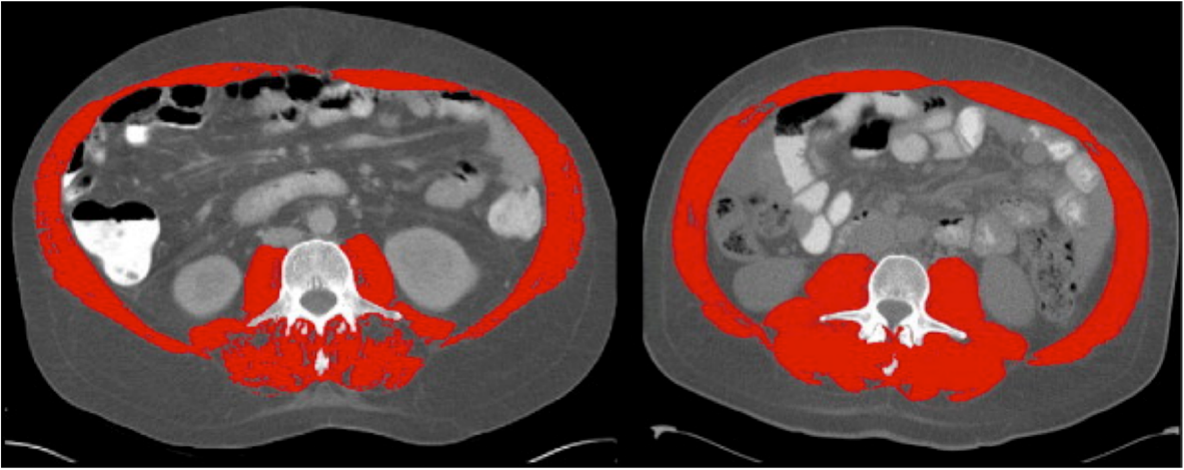
Comparison of sarcopenic and non-sarcopenic computed tomography images at the third lumbar vertebral level. A comparison of two cirrhotic patients with identical BMI (32 kg/m^2^). *Red color* indicates skeletal muscle: rectus abdominis, oblique and lateral abdominal muscles, psoas, and paraspinal muscles. The patient on the *left* is sarcopenic with L3mi of 49.82 cm^2^/m^2^; the patient on the *right* is not sarcopenic with L3mi of 70.8 cm^2^/m^2^. In their study of 112 cirrhotic patients, Montano-Loza et al. used abdominal CT images taken at the third lumbar vertebrae and cutoffs provided by Baumgartner et al. in 1998

Various morphometric measures are used to quantify muscle mass (Table 1), including *appendicular skeletal muscle (ASM) index* (the sum of lean soft-tissue masses for the arms and legs adjusted by height) (Fig. 1) (Baumgartner et al. 1998) and those of core muscles such as total skeletal muscle cross-sectional area at the level of the third lumbar vertebrae (L3mi) (Prado et al. 2008), total psoas area (TPA) (Peng et al. 2011), and psoas density (PD) (Sabel et al. 2011).

Muscle mass has traditionally been measured using BIA or DXA (Chien et al. 2008), but presently, CT and magnetic resonance imaging (MRI) are considered the gold standard methods of assessing body composition in research (Cruz-Jentoft et al. 2010; Bonekamp et al. 2008; Malafarina et al. 2012). CT and MRI are not routinely used in clinical practice for sarcopenia assessment or adiposity assessment, due to concerns about radiation and high cost (Cruz-Jentoft et al. 2010). DXA is inexpensive, most precise for measuring appendicular muscle mass, and exposes patients to minimal radiation but is not portable nor widely available for sarcopenia assessment or adiposity assessment (Bauer and Sieber 2008; Cruz-Jentoft et al. 2010). BIA is an inexpensive, readily reproducible, portable alternative, but results are easily confounded by a number of factors, especially fluid status, and is not preferred for adiposity assessment (Bauer and Sieber 2008).

New image analysis software packages can be used with CT or MRI to streamline segmentation and obtain more consistent body composition measures for sarcopenia assessment. Semi-automated processes like SliceOmatic, NIHImageJ, Analyze, and EasyVision have yielded similar results in terms of reproducibility, while only HippoFat, a fully automated program, has shown to be slightly inferior (Bonekamp et al. 2008). While SliceOmatic segments fastest, NIHImageJ and HippoFat are free. Overall, these software packages have comparable results in analyzing MRI (Bonekamp et al. 2008) and CT images (Irving et al. 2007) but have yet to be incorporated into routine clinical practice.

## Functional implications of SO

In patients >65, SO was associated with a two to three times higher chance of reporting an instrumental activities of daily living (IADL) disability than lean sarcopenic, non-sarcopenic obese, or patients with normal body composition (Baumgartner et al. 2004). While non-sarcopenic obesity and SO were both associated with lower physical activity than non-obese sarcopenic and normal body types, only SO was significantly associated with onset of IADL disability, and low physical activity was not associated with IADL disability in the absence of SO. Thus, SO is considered a worst-case scenario compounding health risks of obesity and depleted lean mass (i.e., malnutrition) (Villareal et al. 2004; Kyle et al. 2005; Roubenoff 2004; Davison et al. 2002), and cancer patients with SO are at higher risk of oncologic treatment-related toxicities and mortality (Tsai 2012).

## Impact of sarcopenia and SO on patient outcome

### Colorectal cancer resection

Sarcopenia has been shown to predict complications and length of stay following colorectal surgery (Lieffers et al. 2012). Moreover, with rising levels of obesity in Western society, sarcopenia may be “hidden beneath” higher BMI.

A 2012 study of stage II–IV colorectal cancer patients undergoing primary colorectal resection found that sarcopenic patients had longer index hospitalization length of stays and post-operative length of stay, especially in those >65 (Table 2) (Lieffers et al. 2012). Sarcopenia was associated with increased infection risk, especially in those >65. Sarcopenic patients required more inpatient rehabilitation, and co-morbidities including cardiac arrhythmias, diabetes, hypertension, and deficiency anemia were more common. In patients >65, sarcopenia independently predicted post-operative infection and need for rehabilitation facility care but did not predict these or longer length of stay in younger patients. Though no explanation was provided, this may be because younger patients are healthier overall (more active, with fewer and less severe conditions and better physiological reserve) and exhibit smaller discrepancies in outcomes. The study provides strong evidence for sarcopenia as a predictor of short-term outcomes in colorectal cancer resection.

**Table 2:** Association between sarcopenia or sarcopenic obesity (SO) and oncological surgery outcomes

Study	Cancer type	Association with short-term oncological outcomes?		Association with long-term oncological outcomes?	
		Sarcopenia	SO	Sarcopenia	SO
Prado (Prado et al. 2008)	Respiratory or GI tract	–	Unclear, but associated with poorer functional status than in non-sarcopenic obese	Yes, independently predicted median survival	Yes, independently predicted survival
Lieffers (Lieffers et al. 2012)	Colorectal (stages II–IV)	Yes, independently predicted post-operative infection risk, longer inpatient rehabilitation, associated with higher risk of obstruction, longer index hospitalization length of stay, longer mean length of stay overall	–	–	–
Sabel (Sabel et al. 2013)	Colon	Yes, independently predicted surgical complications and infectious complications, associated with infectious post-operative complications	Unclear, but SFD is the best predictor of post-operative wound infections, and associated with infectious complications	No, not an independent predictor of disease-free or overall survival	Unclear, but TBF independently predicted disease-free survival
Miyamoto (Miyamoto et al. 2015)	Colorectal (stages I–III)	–	–	Yes, independently associated with disease recurrence rate, overall mortality, cancer-specific mortality, recurrence-free survival, overall survival, cancer-specific survival	–
van Vledder (van Vledder et al. 2012)	Colorectal liver metastases	–	–	Yes, independently predicted disease-free survival and overall survival	–
Dello (Dello et al. 2013)	Colorectal liver metastases	Yes, independently predicted disproportionally small total functional liver volume	Unclear, but fat-free body mass and body surface area independently predicted disproportionally small total function liver volume	–	–
Peng (Peng et al. 2011)	Colorectal liver metastases	Yes, independently predicted major post-operative complications, associated with risk of post-operative complications, overall morbidity risk, longer hospital stays, extended ICU stays	Yes, associated with major post-operative complications, longer hospital stays, extended ICU stays	No, not associated with recurrence-free survival, overall survival or risk of recurrence	No, not associated with overall survival or recurrence-free survival
Lodewick (Lodewick et al. 2015)	Colorectal liver metastases	No, not significantly associated with risk of major post-operative complications, presence of liver surgery-specific composite endpoint^a^ (LSSCEP) items	No, not significantly associated with risk of major post-operative complications, occurrence of one or more of the LSSCEP items	No, not significantly associated with initial hospital length of stay, readmission rates, median disease-free survival, or overall survival	Yes, not predictive of initial hospital length of stay, disease-free survival, or overall survival, but significantly associated with readmission rates
Harimoto (Harimoto et al. 2013)	Liver	Yes, independent predictor of liver dysfunction	–	Yes, independent predictor of overall and recurrence-free survival	–
Voron (Voron et al. 2015)	Liver	No, not associated with severe post-operative complication rate, post-operative mortality or morbidity rates	–	Yes, independently associated with overall and disease-free survival	–
Levolger (Levolger et al. 2015)	Liver	Yes, associated with major post-operative complication (Clavien-Dindo grade ≥IIIa) and treatment-related mortality (within 90 days post-treatment)	–	Yes, associated with overall survival, but not associated with disease-free survival	Yes, associated with shorter median survival
Peng (Peng et al. 2012)	Pancreatic	No, not associated with overall morbidity, major post-operative complications, length of hospital stays, length of ICU stays, or hazard of 90-day death	–	Yes, independent predictor of 3-year mortality	–
Amini (Amini et al. 2015)	Pancreatic	Yes, TPA-sarcopenia not associated with morbidity, but TPV-sarcopenia associated with post-operative complications, major complications, and length of hospital stay. TPV-sarcopenia also independently associated with post-operative complications	Yes, TPV-SO associated with post-operative complications	Yes, TPV-sarcopenia associated with risk of death, and independently associated with risk of death	–
Joglekar (Joglekar et al. 2015)	Pancreatic	Yes, HUAC independently predicted length of stay, ICU stay, major grade III post-operative complications, incidence of any complications. TPA independently predicted length of stay	–	No, HUAC did not predict post-operative overall survival	–

^a^The liver surgery-specific composite endpoint (LSSCEP) is composed of ascites, post-resectional liver failure, bile leakage, intra-abdominal hemorrhage, intra-abdominal abscess, and mortality and was used to assess liver surgery-specific morbidity

A 2015 retrospective study found significant associations between sarcopenia and long-term outcomes (Miyamoto et al. 2015). Sarcopenia was independently associated with higher disease recurrence, shorter recurrence-free survival, and shorter overall survival. Sarcopenia was also independently associated with shorter cancer-specific survival.

Another retrospective study on colon cancer patients undergoing colectomy obtained various morphometric measures to compare their effectiveness as predictors of post-operational outcomes to that of Charlson co-morbidity index scores (Sabel et al. 2013). The single best predictor of any surgical or infectious complications was PD. When considering PD, Charlson scores, age or BMI, or specific co-morbidities were not statistically significant.

After final multivariate models controlled for age and Charlson score, PD was not significantly associated with disease-free or overall survival, but TBF was. The authors concluded that PD-sarcopenia is mainly predictive of non-colorectal cancer deaths in the population and hypothesized that frailty, or immunologic correlates, contributes less to the natural progression of colorectal cancer than it may to other cancers, such as melanoma, which an earlier study by the same authors suggested (Sabel et al. 2011).

A separate cross-sectional study found that SO in patients with solid tumors of the respiratory tract, colon, rectum, or other GI sites was significantly related to worse functional status and independently predicted survival (Prado et al. 2008).

More research is needed to clarify the most effective method of sarcopenia assessment in regard to colorectal cancer resection outcomes. Sarcopenia’s effectiveness as a predictor of long-term outcomes following colorectal cancer resection is unclear, as well as the associations between SO and short- and long-term outcomes.

### Colorectal liver metastases and hepatectomy

Sarcopenia has also been investigated in relation to liver resection. In 2012, a study on patients undergoing colorectal cancer liver metastases determined that sarcopenia was significantly associated with disproportionally small total functional liver volume (TFLV) and impaired short-term outcomes after surgery (Dello et al. 2013). Significant correlation was also found between fat-free body mass and TFLV.

In a separate retrospective analysis, sarcopenia predicted short-term outcomes, but not long-term ones (Peng et al. 2011). Peng et al. found that sarcopenia was strongly univariately associated with increased risk of post-operative complications and overall morbidity, with sarcopenic patients >3 times more likely to develop a major (Clavien grade ≥3) complication (Clavien et al. 2009). Neither TPA nor sarcopenia was predictive of recurrence-free survival, long-term overall survival, or risk of recurrence. While sarcopenic patients had longer hospital stays and higher chances of extended ICU stay (>2 days), SO patients were at even higher risk of both. In addition, SO was associated with an even greater risk of Clavien grade ≥3 complications.

Patients with SO tended to have shorter median overall survival than other patients but, like sarcopenic patients, did not have significant differences in recurrence-free survival compared to the entire cohort. These observations support conclusions from Sabel et al. that perhaps sarcopenia has a less significant impact on the natural progression of colorectal cancer than for other cancers and thus predicts short-term outcomes following surgery in colorectal cancer, but not long-term survival.

**Fig. 2:**
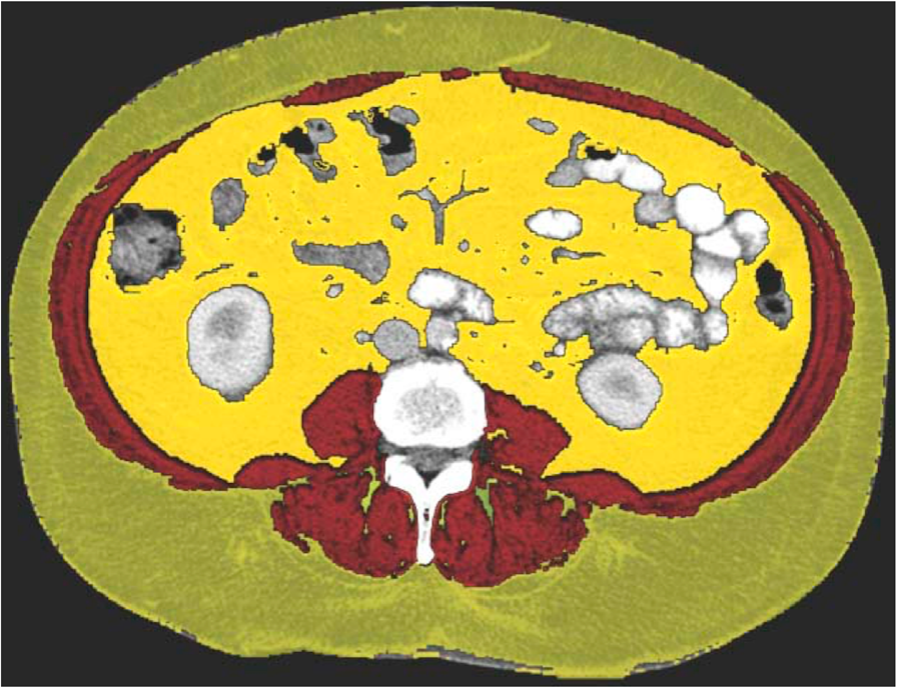
Example segmented L3 computed tomography image for skeletal muscle assessment of patient undergoing hepatic resection. L3mi computed tomogram shows highlighted areas of subcutaneous (*green*) and intra-abdominal fat (*yellow*) and skeletal muscle mass (*red*). van Vledder et al. found a variety of body compositions within their population of 196 patients who underwent hepatic resection for colorectal liver metastases

However, in another retrospective study, sarcopenic patients had shorter median disease-free survival and lower 1-, 3-, and 5-year disease-free survival rates compared to non-sarcopenic patients (Fig. 2) (van Vledder et al. 2012). They also had decreased median overall survival and diminished 1-, 3-, and 5-year overall survival rates. Ultimately, sarcopenia independently predicted worse survival.

Most recently, however, a 2015 study found neither sarcopenia nor SO to be associated with short-term outcomes such as risk of major post-operative complications or morbidity (Lodewick et al. 2015). Moreover, sarcopenia was not significantly associated with long-term outcomes, including readmission rates or disease-free or overall survival. Interestingly, Lodewick et al. observed that sarcopenic patients had significantly higher percentage body fat and investigated SO in relation to outcomes. SO predicted higher readmission rates but no other long-term outcomes.

van Vledder et al.’s findings thus conflict with Peng et al.’s that sarcopenia does not predict long-term survival outcomes, and Lodewick et al. conflict with both on sarcopenia’s association with short- and long-term outcomes. While multiple studies have supported an association between sarcopenia and short-term outcomes following hepatectomy of colorectal liver metastases, uncertainty remains about this association and surrounding sarcopenia’s relation to long-term outcomes.

### Hepatocellular carcinoma and hepatectomy

A 2013 retrospective study on partial hepatectomy in patients with hepatocellular carcinoma (HCC) noted that there is currently no objective, accessible, accurate measure of a patient’s condition before undergoing hepatectomy (Harimoto et al. 2013). While the American Society of Anesthesiologists’ (ASA) grading estimates functional status, it is subjective (Makary et al. 2010). Child-Pugh scores are not always reliable metrics of hepatic reserve, and Model for End-Stage Liver Disease (MELD) scores quantify mortality risk in end-stage liver disease and organ allocation processes (Teh et al. 2005). In this study, sarcopenia independently predicted overall and recurrence-free survival following surgery (Harimoto et al. 2013). As serum albumin levels were significantly lower and indocyanine green dye retention rate at 15 min (ICGR15) values higher in sarcopenic patients than in non-sarcopenic patients (conveying less effective hepatic removal of synthetic dye from plasma, worse hepatic functional reserve and ability to regenerate), sarcopenia was significantly associated with liver dysfunction. This study strongly supported sarcopenia as a predictor of short- and long-term outcomes following hepatectomy.

Subsequent studies disagreed. A 2015 retrospective study found that while sarcopenia was independently associated with overall and disease-free survival, it was not with short-term outcomes (Voron et al. 2015). Post-operative mortality, morbidity, and major complication rates were higher in sarcopenic than non-sarcopenic subjects but not statistically significantly so.

A separate 2015 retrospective study reaffirmed initial findings from Harimoto et al. (Levolger et al. 2015). Unlike Voron et al., Levolger et al. discovered that sarcopenia was associated with short-term outcomes such as increased risk of major post-operative complications and treatment-related mortality (death within 90 days of treatment). It was also associated with some long-term outcomes, like shorter overall survival, but not disease-free survival.

Further research needs to evaluate the effectiveness of sarcopenia as a predictor of short-term outcomes following hepatectomy for hepatocellular carcinoma and clarify the extent to which it is a useful predictor of long-term outcomes.

Additionally, Voron et al. reported a wide range of BMI across sarcopenic subjects and emphasized the limited practicality of BMI in sarcopenia determination without CT analysis. Three subjects with identical BMI had varying L3mi values and sarcopenic status. Similarly, Levolger et al. noted the prevalence of sarcopenia in various body compositions and found that those overweight or obese and sarcopenic have significantly shorter median survival rates than non-sarcopenic obese or overweight. These findings highlight the necessity of using CT to separately screen for sarcopenia, which is convenient, as many hepatocellular carcinoma patients already undergo routine imaging (Voron et al. 2015).

### Pancreatic adenocarcinoma and pancreatic resection

Recent literature has found that factors such as co-morbidity, anemia, and sarcopenia may affect post-operative outcome as significantly as adjuvant oncological treatments (Fearon et al. 2013). Considerable variation in 30-day morbidity and mortality following cancer surgery and in long-term outcomes between countries supports the idea that factors outside traditional multidisciplinary team consideration may be important determinants of outcome (Baili et al. 2008; van Gijn et al. 2010).

A retrospective study on patients undergoing resection of pancreatic adenocarcinoma found that sarcopenia was an objective measure of patient frailty strongly predictive of long-term outcomes, independent of tumor-specific factors (Peng et al. 2012). Sarcopenia was not associated with short-term outcomes such as risk of overall morbidity or major complications, median ICU stays, or length of hospital stays. It was associated with increased risk of 3-year mortality. This relationship suggests that perhaps individual patient characteristics have a stronger impact on long-term outcomes of pancreatic resection than tumor-specific factors do.

However, more recent retrospective studies using volumetric or density psoas metrics found stronger associations between sarcopenia and short-term outcomes. In 2015, a retrospective study revealed stronger associations between total psoas volume (TPV)-sarcopenia and short-term outcomes than with TPA-sarcopenia (Amini et al. 2015). While TPA-sarcopenia was significantly related to risk of morbidity, TPV-sarcopenia was significantly associated with risk of any post-operative complication, risk of major complications, and length of hospital stay. Moreover, TPV-sarcopenia independently predicted risk of death. TPV-SO was also associated with higher risk of post-operative complications compared to non-sarcopenic obese.

The same year, Joglekar et al. investigated the Hounsfield unit average calculation (HUAC), a measure of psoas muscle density and fatty infiltration, along with TPA (Joglekar et al. 2015). Like TPV-sarcopenia, they found that HUAC-sarcopenia was associated with more short-term outcomes than TPA-sarcopenia. TPA-sarcopenia was associated with longer length of hospital stay, while HUAC-sarcopenia was associated with longer length of hospital stay, length of ICU stay, risk of any post-operative complication, and risk of major complications. One point of contrast between Joglekar et al. and Amini et al. was that unlike TPV-sarcopenia, HUAC-sarcopenia did not strongly predict long-term outcomes.

These findings reiterate the importance of identifying the most clinically significant assessment methods of sarcopenia before determining its utility as a predictor. Amini et al. determined that volumetric standards for sarcopenia like TPV might be more clinically useful as predictors of cancer surgery outcomes than cross-sectional area measures such as TPA. Similarly, a definition based on muscle density forecasted short-term outcomes better than TPA (though not long-term outcomes) (Joglekar et al. 2015).

## Strategies to minimize the impact of sarcopenia and SO

While sarcopenia assessment can help identify cancer surgery patients at risk of worse outcomes, it is important to note that sarcopenia and SO themselves are modifiable. The most effective interventions to date are physical exercise and adequate nutritional protein intake (Deutz et al. 2014; Bauer et al. 2013). Pharmacological therapies for sarcopenia including inhibitors of myostatin, testosterone, selected androgen receptor modulators, ghrelin agonists, and angiotensin-converting enzyme (ACE) inhibitors have been evaluated, but preliminary trials have found that they are less effective than postulated (Molfino et al. 2016; Morley et al. 2014). In older adults vulnerable to functional decline, moderate-intensity physical activity significantly reduces risk of onset of major mobility disability compared to health education and, to a larger degree, risk of persistent mobility disability (Pahor et al. 2014). Since the primary outcome of sarcopenia is mobility disability, a proven intervention from the mobility disability literature, moderate physical activity, is a promising intervention for non-aging sarcopenic patients as well. In addition, relative sarcopenia in obese elderly was significantly ameliorated by diet regimens, exercise programs, or their combination (Villareal et al. 2011). Fat mass decreased under diet or exercise programs or their combination, but lean body mass decreased less under diet-exercise than with only diet and increased under an exercise program. A diet and exercise program yielded the greatest improvements in physical performance and the most consistent changes in strength, balance, and gait and thus may be an important therapy for SO subpopulations.

## Conclusions

The impact of sarcopenia on post-operative oncologic outcomes and its usefulness as a predictor is still unclear and often conflicting, particularly in the case of hepatectomy for colorectal liver metastases and hepatectomy for hepatocellular carcinoma. However, sarcopenia is promising as a predictor of short-term outcomes following colorectal cancer surgery and long-term outcomes following hepatectomy for hepatocellular carcinoma. SO is promising as a predictor of long-term outcomes following hepatectomy for hepatocellular carcinoma and short-term outcomes following pancreatic resection. The other associations need additional clarification, as studies provided inconsistent results.

There remains considerable variation in definition, cutoffs, and assessment methods for sarcopenia and SO, which makes translation to clinical practice complicated. Recent criteria incorporating both grip strength and appendicular skeletal muscle allow more clinically meaningful associations with long-term outcomes and are preferred to definitions excluding a functional component (Studenski et al. 2014; McLean et al. 2014). Though ramifications have been explored in geriatric populations, sarcopenia assessment may be more easily assimilated into clinical practice for cancer surgery patients, as routine imaging is already completed for these populations. Semi-automated segmentation software also promises to play a major role in streamlining sarcopenia assessment integration into routine practice.

Sarcopenia and SO may predict short- and long-term outcomes, and effective identification of patients at risk of poorer outcomes from cancer surgery allows for tailored interventions. As clinicians become increasingly aware of this subtle form of malnutrition, addressing sarcopenia preoperatively can optimize outcomes for at-risk patients.

### Future directions

Further research is needed to reach consensus regarding the ideal manner of assessing sarcopenia in order to predict outcomes after various cancer surgeries and across cancers in a broad clinical sense. The prevalence and impact of SO in various cancer surgeries requires further examination. Risk of mortality associated with SO for all types of patients should be further evaluated (Prado et al. 2008; Batsis et al. 2013). It remains unclear whether the added risk from SO is the sum of the individual impacts of obesity and sarcopenia or whether these conditions further interact with each other. Finally, current interventions for sarcopenia and SO have not been robustly verified, due to challenges from risk of adverse reactions when dealing with sarcopenic populations (Cesari M 2016). Furthermore, these interventions have yet to be tested specifically with regard to cancer surgery patients.

## Abbreviations

ACE: Angiotensin-converting enzyme
ASA: American Society of Anesthesiologists
ASM: Appendicular skeletal muscle
BIA: Bioelectric impedance analysis
BMI: Body mass index
CT: Computerized tomography
DXA: Dual x-ray energy absorptiometry
GI: Gastrointestinal
HCC: Hepatocellular carcinoma
HUAC: Hounsfield unit average calculation
IADL: Instrumental activities of daily living
ICGR15: Indocyanine green dye retention rate at 15 min
L3mi: Total skeletal muscle cross-sectional area at the level of the third lumbar vertebrae
LSSCEP: Liver surgery-specific composite endpoint
MELD: Model for End-Stage Liver Disease
MRI: Magnetic resonance imaging
PD: Psoas density
SD: Standard deviation
SFD: Subcutaneous fat distance
SO: Sarcopenic obesity
TBF: Total body fat
TFLV: Total functional liver volume
TPA: Total psoas area
TPV: Total psoas volume
WC: Waist circumference
